# Non-invasive assessment of NAFLD as systemic disease—A machine learning perspective

**DOI:** 10.1371/journal.pone.0214436

**Published:** 2019-03-26

**Authors:** Ali Canbay, Julia Kälsch, Ursula Neumann, Monika Rau, Simon Hohenester, Hideo A. Baba, Christian Rust, Andreas Geier, Dominik Heider, Jan-Peter Sowa

**Affiliations:** 1 Department of Gastroenterology, Hepatology, and Infectious Diseases, Otto-von-Guericke University Magdeburg, Magdeburg, Germany; 2 Department of Gastroenterology and Hepatology, University Hospital, University Duisburg-Essen, Essen, Germany; 3 Institute for Pathology, University Hospital, University Duisburg-Essen, Essen, Germany; 4 Department of Mathematics and Computer Science, University of Marburg, Marburg, Germany; 5 Division of Hepatology, Department of Internal Medicine II, University Hospital Würzburg, Würzburg, Germany; 6 Department of Medicine II, University Hospital, LMU Munich, Munich, Germany; 7 Center for Nutritional Medicine and Prevention, Department of Medicine I, Hospital Barmherzige Brüder, Munich, Germany; Medizinische Fakultat der RWTH Aachen, GERMANY

## Abstract

**Background & aims:**

Current non-invasive scores for the assessment of severity of non-alcoholic fatty liver disease (NAFLD) and identification of patients with non-alcoholic steatohepatitis (NASH) have insufficient performance to be included in clinical routine. In the current study, we developed a novel machine learning approach to overcome the caveats of existing approaches.

**Methods:**

Non-invasive parameters were selected by an ensemble feature selection (EFS) from a retrospectively collected training cohort of 164 obese individuals (age: 43.5±10.3y; BMI: 54.1±10.1kg/m^2^) to develop a model able to predict the histological assessed NAFLD activity score (NAS). The model was evaluated in an independent validation cohort (122 patients, age: 45.2±11.75y, BMI: 50.8±8.61kg/m^2^).

**Results:**

EFS identified age, γGT, HbA1c, adiponectin, and M30 as being highly associated with NAFLD. The model reached a Spearman correlation coefficient with the NAS of 0.46 in the training cohort and was able to differentiate between NAFL (NAS≤4) and NASH (NAS>4) with an AUC of 0.73. In the independent validation cohort, an AUC of 0.7 was achieved for this separation. We further analyzed the potential of the new model for disease monitoring in an obese cohort of 38 patients under lifestyle intervention for one year. While all patients lost weight under intervention, increasing scores were observed in 15 patients. Increasing scores were associated with significantly lower absolute weight loss, lower reduction of waist circumference and basal metabolic rate.

**Conclusions:**

A newly developed model (http://CHek.heiderlab.de) can predict presence or absence of NASH with reasonable performance. The new score could be used to detect NASH and monitor disease progression or therapy response to weight loss interventions.

## Introduction

Non-alcoholic fatty liver disease (NAFLD) is a growing public health problem worldwide [1]. NAFLD represents the liver manifestation of the metabolic syndrome and the underlying origin seems to be excess lipid accumulation. Development and mechanisms are complex and not completely clear [2–4]. By now, NAFLD is a major cause of liver-related morbidity and mortality [1], without apparent serological signs of injury [5]. NAFLD represents a serious health concern and will amount to significant burden on health care systems [1,6]. One problem among many regarding NAFLD is stratification of patients by risk of progression, as substantial morbidity and mortality arise from cardiovascular and other causes [7,3,8]. Another problem is that serum parameters used to assess severity of other chronic liver diseases remain well within normal ranges for most NAFLD patients [9,10].

The diagnostic gold standard for NAFLD assessment remains liver biopsy, a potentially painful and harmful procedure for the patient. In a liver biopsy 5% lipid content can be detected and, more importantly, inflammation, hepatocellular ballooning, and fibrotic alterations can be assessed [11]. These are required to diagnose non-alcoholic steatohepatitis (NASH). While accumulation of lipids in the liver can be assessed by imaging techniques [12] non-invasive tests cannot accurately detect inflammation and hepatocellular ballooning. Due to the invasive nature and the required effort to take a liver biopsy, it is not suited to monitor disease progression or regression. Thus, development of non-invasive scoring systems to predict severity of NAFLD, either based on (advanced) fibrosis or NASH, is a vital field of clinical research [13–20]. Despite several available scores and biomarkers, no ideal set of markers has been agreed upon. Many scores fail validation in independent cohorts, require a relatively large number of markers, too complicated to handle in clinical routine, or are nontransparent, making validation in independent cohorts impossible [19,21,22]. Objective biomarker panels for assessment and monitoring of NAFLD or NASH are still urgently needed to improve clinical routine and research [23].

In the current study, we initially aimed at comparing currently available scores [13–16,20] for the non-invasive detection of NASH in morbidly obese patients. Histological separation of NAFL and NASH was based on the non-alcoholic steatohepatitis activity score (NAS) [24], as this is still the more widely applied and known system. As most tested scores were unable to predict NASH or fibrosis with reasonable accuracy, we aimed for a new non-invasive score for separation of NAFL from NASH. An ensemble feature selection approach (EFS) [25] identified biomarkers associated with NAS from routine markers, adiponectin and the apoptosis marker M30 [26,27]. A logistic regression model based on these biomarkers was built and validated in an independent study cohort.

## Patients and methods

### Ethics statement

The study protocol conformed to the ethical guidelines of 1975 Declaration of Helsinki and was approved by the Institutional Review Board (IRB; Ethik-Kommission der Medizinischen Fakultät der Universität Duisburg-Essen; Germany; 15-6356-BO). Due to the retrospective nature of the training study the IRB waived the need for written informed consent. All procedures adhered to the Declaration of Helsinki and the requirements of the IRB.

The protocol of the prospectively recruited validation study cohort was approved by the local Ethics Committee and the Review Board of the University Hospital Würzburg (Ethik-Kommission der Medizinischen Fakultät der Universität Würzburg; AZ96/12). Written informed consent was obtained from all participants.

All individuals participating in the (non-invasive) lifestyle intervention study were prospectively collected and gave written informed consent to the study protocol.

All authors had access to the study data and reviewed and approved the final manuscript.

### Study design and sample acquisition

The training cohort (University Hospital Essen) consisted of 164 obese patients with histologically confirmed NAFLD (NAS 1–8; Table 1), who underwent bariatric surgery. Patients were eligible for the retrospective study, when liver histology was available and a NAS of at least 1 was diagnosed by a pathologist. Patients received dietary and exercise counselling for 6 months prior surgery, no calorie restriction was imposed. A blood sample was collected for assessment of serum derived factors on the day of surgery (prior surgery) and liver tissue was sampled during bariatric surgery. Detailed demographic and clinical information of this cohort is given in Table 1. All data shown were recorded on the day of surgery.

**Table 1:** Basic clinical and demographic data of the training and evaluation cohorts.

Parameter	Training cohort (UH Essen)	Evaluation cohort (UH Würzburg)
N	164	122
Female / male	124 / 40 (76% / 24%)	96 / 26 (79% / 21%)
Age^1^	43.5±10.3 y	45.2±11.75 y
BMI^1^	54.1±10.1 kg/m^2^	50.8±8.6 kg/m^2^
ALT^1^	33.6±21.4 U/l	-
AST^1^	28.7±15.0 U/l	-
GGT^1^	49.5±99.6 U/l	58.9±73.81 U/l
HbA1c^1^	6.2±1.5%	6.1±1.2%
Fasting Glucose^1^	114.9±56.9 mg/dl	-
Total cholesterol^1^	199.7±40.4 mg/dl	-
M30^1^	321.7±261.1 U/l	349.3±372.6 U/l
M65^1^	607.5±614.7 U/l	-
Adiponectin^1^	3.9±2.8 μg/ml	4.4±2.9 μg/ml
NAS^2^	4 (1–8)	3 (1–8)
Fibrosis^2^	2 (0–4)	-
Apri Score^2^	0.2 (0.1–1.1)	-
BARD Score^2^	2 (1–3)	-
NAFLD fibrosis Score^2^	0.34 (-2.6–3.1); n = 49	-
CHeK^2^	5.8 (-2.7–102.0)	6.7 (-2.9–51.97)
Distribution of NAS results	1: 22 (13%)	1: 15 (12%)
	2: 17 (10%)	2: 25 (20%)
	3: 35 (21%)	3: 23 (19%)
	4: 29 (18%)	4: 27 (22%)
	5: 27 (16%)	5: 17 (14%)
	6: 16 (10%)	6: 9 (7%)
	7: 12 (7%)	7: 5 (4%)
	8: 6 (4%)	8: 1 (1%)
Histological NAFL / NASH	NAFL: 103 (62%) NASH: 61 (38%)	NAFL: 90 (74%) NASH: 32 (26%)
Distribution of fibrosis results	0: 25 (16%)	
	1: 41 (25%)	
	2: 94 (57%)	
	3: 3 (2%)	
	4: 1 (0.6%)	

^1^Continuous parameters are presented as mean ± standard deviation.

^2^Categorical parameters and non-parametrical variables are presented as median (range).

The validation cohort was recruited prospectively at the University Hospital Würzburg (Division of Hepatology), including 122 patients. The majority of these patients underwent bariatric surgery (n = 105). Detailed demographic and clinical information of this cohort is given in Table 1. Patients were eligible when all parameters used in the newly generated score were complete and histological assessment of the NAS was available.

Pathologists assessed the NAS prior development of the new score and were thus blinded to the results of the new score. While during training the NAS as reference was required to be unblinded, calculation of the new score in the validation cohort was performed blinded to the NAS.

To assess a possible use of the new score for monitoring of treatment, data from a cohort recruited for lifestyle intervention at the University of Munich (Department of Medicine II) were applied [28]. The whole study cohort consisted of 152 patients. For the present analysis 38 patients with complete datasets to generate the new score at start and end of the study were selected.

### Dataset and statistics

The training dataset included the socio-demographic parameters sex, age, height, weight, and BMI, as well as the serum parameters ALT, AST, AST/ALT ratio, GGT, albumin, triacylglycerols, total cholesterol, fasting blood sugar, HbA1c, thrombocyte count, total (M65) and caspase-cleaved serum CK-18 (M30), and adiponectin. In liver tissue steatosis, ballooning, lobular inflammation, and fibrosis were assessed by two independent pathological reviewers (JK, HAB). From these available parameters, the presence of NAFL or NASH was determined and the NAS, the APRI score (http://www.labor-limbach.de/AST-Thrombozyten-Rat.391.0.html?&no_cache=1&L=0) [20], the BARD score (http://gihep.com/calculators/hepatology/bard/) [16], the NAFLD fibrosis score (http://nafldscore.com/) [14], the Palekar Score [13], and the Gholam score [15] were calculated. Calculation of the Sumida Score [18], SteatoTest, and ActiTest / NASHTest [19] could not be performed based on the available data.

The validation dataset included age, HbA1c, GGT, M30, adiponectin, sex, BMI, and NAS. Statistical data analyses were performed with R (http://www.r-project.org/) and Prism Version 5 (Graphpad Inc., La Jolla, CA, USA). All data are presented as mean ± standard error of the mean (SEM) unless specified otherwise. Correlation analysis was performed using Spearman’s rank correlation coefficient.

### Importance analysis and predictive modeling

Importance analysis of the biomarkers were carried out with EFS with default settings [25] using the web-interface (http://efs.heiderlab.de). EFS aggregates eight different feature selection methods and provides a quantitative ranking of the features [29]. EFS has been shown to compensate biases of single feature selection methods, overcoming instability and unreliability of biomarker discovery approaches.

The biomarkers with the highest ranks identified by EFS, were used for subsequent model development by logistic regression. The *stats* package of R with standard settings was used. For evaluation of the model performance a 10-fold cross-validation scheme was used and the Receiver Operation Characteristics (ROC) curve and the corresponding Area under the Curve (AUC) were calculated with pROC [30]. The 95% CI was computed with 2000 stratified bootstrap replicates.

## Results

### Proposed non-invasive scores do not correlate with histological assessment of NAFLD severity

To assess performance of existing non-invasive scores, we calculated correlations between APRI, BARD, Gholam’s score, NAFLD fibrosis score (NFS), and Palekar’s score and NAS, and fibrosis, respectively. Of note, APRI and NFS were developed to assess severity of fibrosis/cirrhosis, but have been tested for assessment of NASH by other groups previously. Only the APRI (r = 0.3269, p < 0.001) and Gholam’s scores (r = 0.3670, p < 0.0001) were significantly, positively correlated with NAS (S1A and S1B Fig), while other scores were not (S1C–S1E Fig). ROC curves were built for each score to differentiate between NAFL (NAS ≤ 4) or NASH (> 4) as additional measure of performance. APRI (AUC: 0.6523, p = 0.001) and Gholam (AUC: 0.7001, p < 0.0001; S2A and S2B Fig) were able to separate between NAFL and NASH. No significant correlations were found for the scores with the grade of fibrosis (S3 Fig). Demographic and clinical data of this cohort are given in Table 1.

### A new score with unbiased parameter selection reaches high accuracy to predict NASH in the training cohort

To improve non-invasive classification of NAFL *versus* NASH, all available markers were evaluated by EFS. The markers age, HbA1c, GGT, adiponectin, and M30, were used as input for a logistic regression model. Logistic regression models are easy to interpret, have been widely applied and have very high acceptance among physicians in clinical practice [31]. The model was evaluated using a 10-fold cross-validation. The model is publicly available at http://CHek.heiderlab.de.

**Fig 1:**
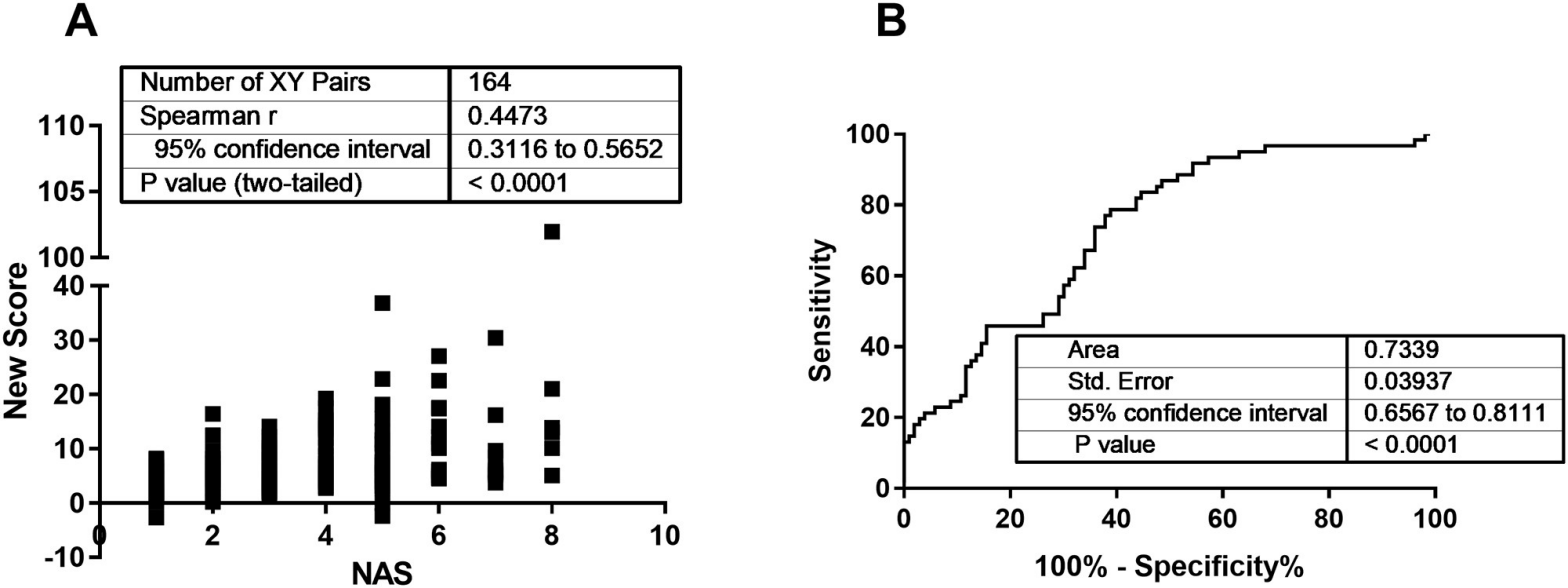
Performance of the new score within the training cohort. Based on available non-invasive parameters in an obese cohort of 164 patients a new score was generated. This score correlated strongly with the NAS (A), assessed by two independent pathologists, in this cohort. For separation between NAFL (NAS ≤ 4) and NASH (NAS > 4) an AUC of 0.73 was achieved (B).

The predicted scores are strongly correlated with NAS (r = 0.4473; p < 0.0001; Fig 1A) and the model reached an AUC of 0.7339 (95% CI: 0.6567–8111; p < 0.0001; Fig 1B). The AUC of the model was significantly higher compared to all other tested scores. While no correlation was found for the new score and fibrosis stage, the AUC of a ROC curve to separate moderate from advanced fibrosis reached 0.8117 (95% CI: 0.6964 to 0.9271; p = 0.03; advanced fibrosis n = 4).

### The new score achieves high accuracy in an independent validation cohort

**Fig 2:**
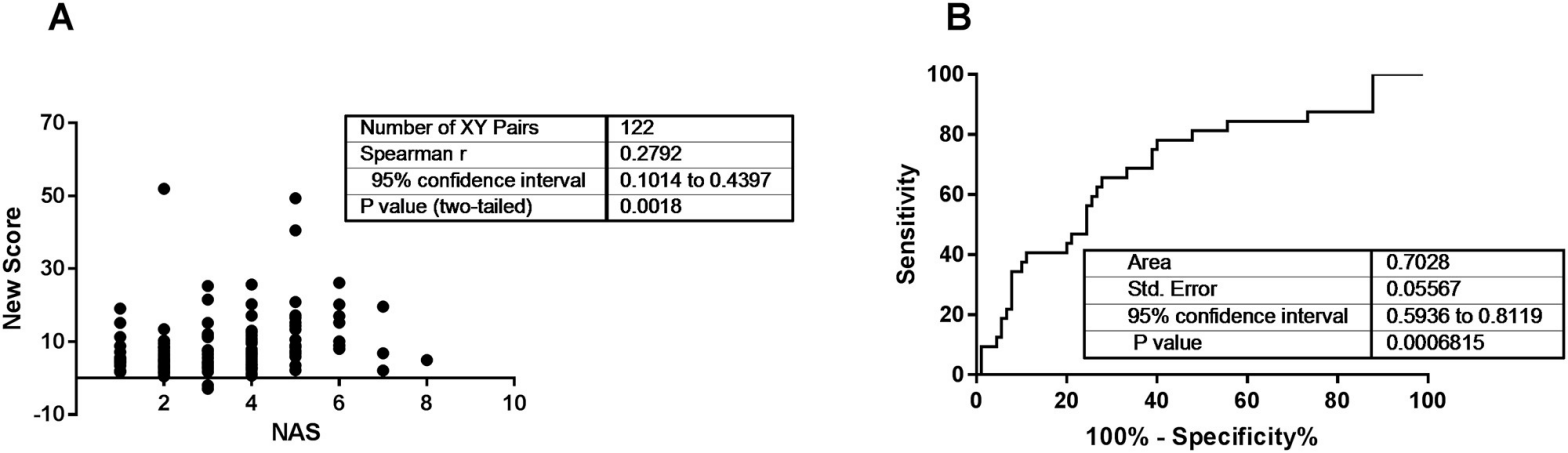
The new score achieves reasonable performance in a validation cohort. The newly generated score was evaluated in an independent cohort from the University Hospital Würzburg (A, B) with 122 patients. For the validation cohort the new score achieved a Spearman correlation coefficient with the NAS of 0.28 (A) and was able to differentiate between NAFL (NAS ≤ 4) and NASH (NAS > 4) with an AUC of 0.7 (B).

In the validation cohort (n = 122, see Table 1) the new model showed performance similar to the training set. The score is significantly correlated with NAS (r = 0.2792; p = 0.002; Fig 2A) and the model reached an AUC of 0.7028 (p = 0.0007; Fig 2B). In addition, the model was applied to separate NAFL and NASH according to the classification by SAF, achieving slightly lower AUC in the training cohort (S4 Fig). The new model can predict severity of NAFLD with reasonable performance.

### The new score can be applied to monitor therapy response in patients with metabolic alterations

Due to the fact that the new model was able to separate NAFL and NASH in an independent cohort with good accuracy, we explored, whether it would be applicable for monitoring response to weight loss therapy. To this end, a cohort of patients participating in a lifestyle intervention program to treat morbid obesity for one year, based on the current guideline of the German Association of Obesity [32], was analyzed (Table 2) [28]. No liver biopsies were available in this cohort. Complete datasets for calculating the new score at start and end of the study were available for 38 patients. The treatment regime led to weight loss in all patients and an overall improvement of metabolic parameters and health indices in the majority of patients. In parallel, a drop of the new score was observed in 60% of the patients (26 with reduced score, 17 with increased score). Mean reduction of the new score was 1.53 points. This would correspond to a reduction in NAS by 1 point for seven patients, including one case of hypothetical resolution from NASH (NAS = 5) to NAFL (NAS = 4).

**Table 2:** Basic clinical and demographic data of a subgroup of the lifestyle intervention study cohort.

Parameter	T0 (start of study)	T52 (end of study)
N	38	Unchanged
Female / male	27 / 11 (71% / 29%)	Unchanged
Age	42.1 ± 10.8 y	43.1 ± 10.8 y
BMI	41.9 ± 6.4 kg/m^2^	33.9 ± 5.9 kg/m^2^
ALT	33.0 ± 18.8 U/l	22.4 ± 9.3
AST	25.9 ± 10.4 U/l	22.6 ± 5.9
GGT	38.3 ± 47.4 U/l	28.5 ± 32.0 U/l
HbA1c	5.6 ± 0.7	5.3 ± 0.6
HDL	52.7 ± 14.5	60.5 ± 16.9
LDL	127.6 ± 40.3	112.3 ± 34.2
Adiponectin	7.0 ± 4.9	8.4 ± 5.8
M30	212.8 ± 220.1 U/l	220.1 ± 229.4
CHeK	2.0 (-3.8–21.4)	1.7 (-6.4–11.0)
Presence of steatosis in ultrasound	31 (82%)	13 (46%; known for 28)

#### The new score indicates efficacy of weight loss therapy to restore metabolic health

**Fig 3:**
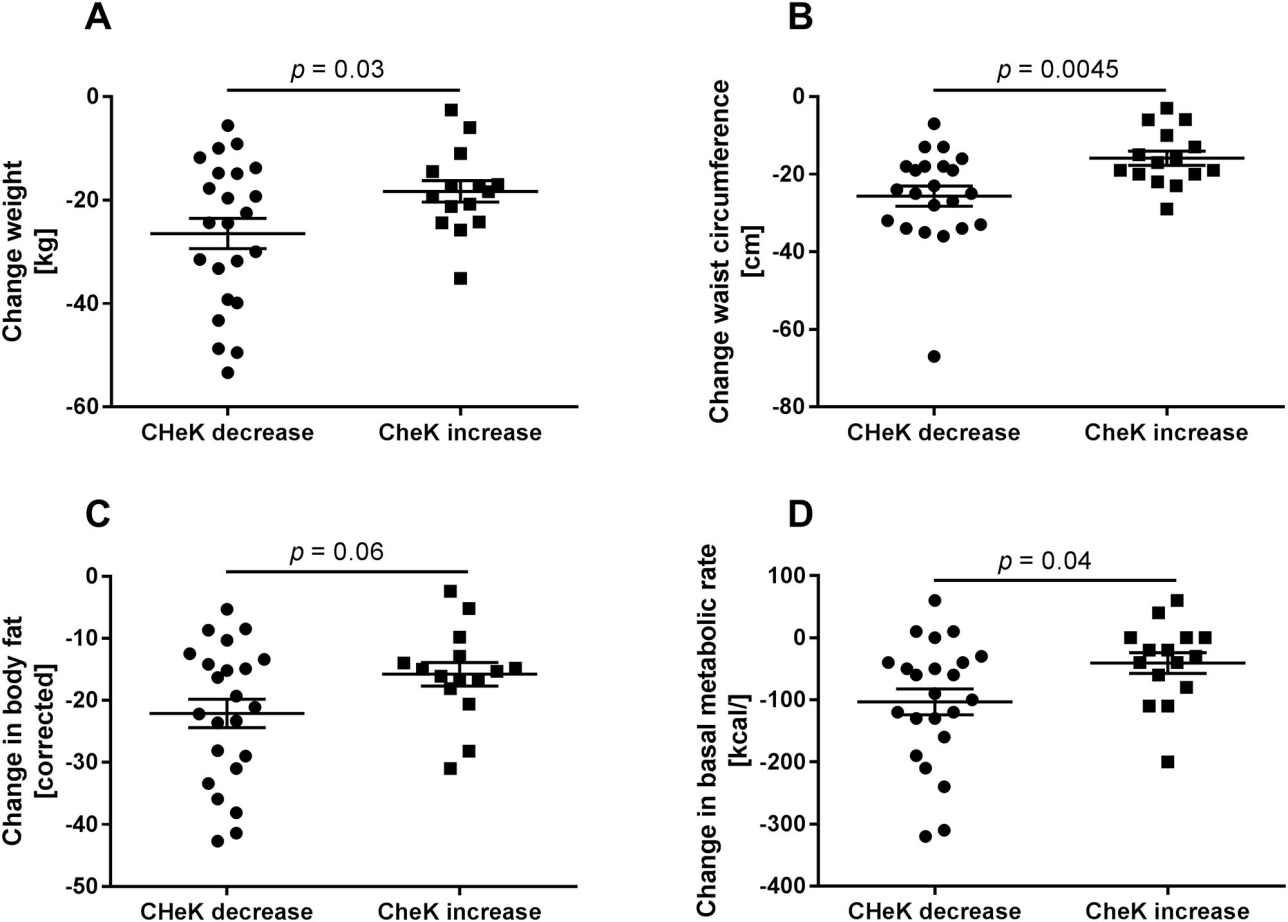
Differences in response to weight loss therapy indicated by the new score. In a subgroup of 38 patients receiving weight loss therapy for 1 year (Tables 2 and 3) only 23 patients (60%) exhibited a reduction of the newly generated score, while weight and most other metabolic health indicators were improved. Individuals with an increase of the score (15 patients, 40%) during study duration had significantly lower absolute weight loss (A), lower reduction of waist circumference (B), nominally lower reduction in body fat content (C), and a significantly less pronounced impairment of basal metabolic rate (D).

**Fig 4:**
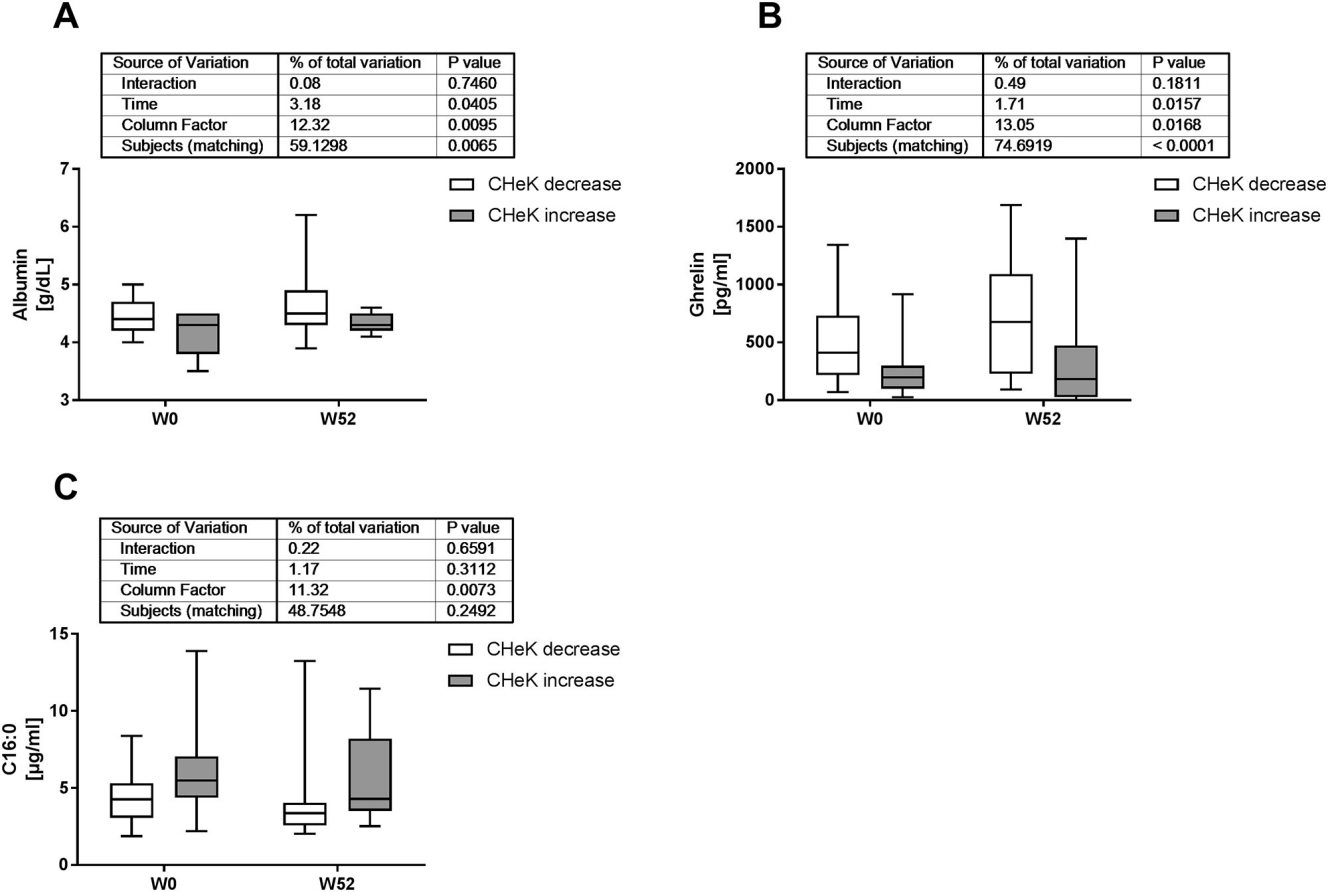
The new score indicates liver and adipose tissue function as possible determinants for weight loss efficiency. 38 patients receiving weight loss therapy for 1 year (Tables 2 and 3) were grouped by either reduction (23 patients) or increase (15 patients) of the new score. In the group with decreased score values at end of the study duration we found significantly higher serum albumin (A) and ghrelin (B) concentrations at start and end of the study. Conversely, concentration of the unsaturated fatty acid palmitic acid (C16:0) was significantly lower at both time points in patients with decrease of the score during weight loss therapy.

All treated patients exhibited weight loss and at least mild improvements of the clinical situation. Since the new score was reduced in only 60% of the patients, correlation analyses were performed to identify markers which might explain this discrepancy. Significant correlations of the new score at start (T0) and end (T52) of the study are given in S1 and S2 Tables. Interestingly, the new score is correlated to almost all metabolic and liver related health indicators, including body composition and basal metabolic rate at both time points. We then grouped patients by either a decrease of—3.8 [(-10.32)—(-0.05)] points or increase of 2.5 (0.13–9.7) points of the score during the treatment duration. Basic parameters of these subgroups did not differ at T0 (Table 3) but suggested a slightly better liver related health condition for individuals with an increase of the new score. At T0 the new score was significantly higher in patients with stronger reduction of the new score. Patients with decreased score exhibited significantly higher absolute weight loss (Fig 3A), and stronger reduction of waist circumference (Fig 3B) at the end of the study duration. While reduction of corrected body fat proportion (NutriPlus Version 5.4.1, Data input, Poecking, Germany) was not significantly different (Fig 3C) the change of basal metabolic rate was higher in individuals with reduced score (Fig 3D). In addition, serum albumin and the gastrointestinal hormone ghrelin were significantly higher in patients with reduced score at both time points (T0 and T52; Fig 4A and 4B). Conversely, serum palmitic acid (C16:0; Fig 4C) was significantly lower in patients with reduced score at T0 and T52. Fatty liver index or NAFLD fibrosis score did not differ between patients with decrease or increase of the new score (not shown). When applied as monitoring tool the new score can indicate efficacy of weight loss treatment for restoration of metabolic health.

**Table 3:** Basic clinical and demographic data of a subgroup of the lifestyle intervention study cohort separated by CHeK alterations during study course (data from T0).

Parameter	CHeK decrease	CHeK increase
N	23	15
Female / male	15 / 8 (65% / 35%)	12 / 3 (80% / 20%)
Age	42.0 ± 11.0 y	42.3 ± 11.0 y
BMI	42.5 ± 6.9 kg/m^2^	40.9 ± 5.6 kg/m^2^
ALT	34.4 ± 17.8 U/l	30.8 ± 20.8
AST	26.4 ± 7.7 U/l	25.3 ± 14.1
GGT	45.5 ± 59.0 U/l	27.2 ± 16.2 U/l
HbA1c	5.6 ± 0.8	5.7 ± 0.6
HDL	54.0 ± 13.5	50.7 ± 16.2
LDL	128.2 ± 43.4	126.5 ± 36.6
Adiponectin	6.4 ± 4.9	8.0 ± 4.8
M30	243.8 ± 271.2 U/l	165.3 ± 91.9
CHeK	3.9 (-2.9–21.4)	0.8 (-3.8–6.8)*
Presence of steatosis in ultrasound	18 (78%)	13 (87%)

*: *p* vs. patients with CHeK decrease < 0.05

## Discussion

The non-invasive assessment and monitoring of NAFLD is a complex problem and has been reviewed outstandingly [23,33,34]. In brief, histological evaluation still remains the gold standard, but is limited by sampling error and inter- as well as intra-observer variance in assessment [35]. Moreover, liver biopsy procedures needed for histological assessment are time-consuming, require trained experts, and pose a risk for the patient [36,37]. Magnetic resonance (MR) imaging or MR elastography might be more exact and less dangerous compared to biopsy, but are also costly and time-consuming, making a screening or monitoring inefficient [38]. Other non-invasive methods, as ultrasound or transient elastography lack sensitivity and accuracy. No known single non-invasive parameter achieves sufficient sensitivity and specificity to discern NAFL from NASH, let alone reflect nuanced NAS-based grading [39]. The current consensus seems that more exact and easy accessible biomarker panels are warranted for research and disease monitoring [23,34].

Due to the described diagnostic dilemma many scores and methods for non-invasive assessment of NAFLD have been proposed in various settings. In the present study, we evaluated previously published scoring systems in a specific cohort, to identify the ideal one for our purposes. Unfortunately, few of the scores correlate with NAS or fibrosis score in our cohort. The best performing Gholam score includes presence of diabetes as a dichotomous variable, prohibiting disease or therapy monitoring, in particular for short time frames. Separation of NAFL versus NASH is only possible with the APRI score, initially developed to predict advanced fibrosis in NASH, with a suboptimal AUC of 0.65. Another disadvantage of many scores is a dichotomous separation of disease entities: non-NASH vs. NASH, no/mild fibrosis vs. advanced fibrosis. This is sufficient in clinical settings to decide further diagnostics or therapy for an individual patient. However, it does not reflect the underlying biology, where a more nuanced spectrum exists. Non-invasive scores to predict severity of NAFLD or fibrosis have limited performance in independent settings, as shown in the current study.

The unsatisfactory performance of existing scores in our setting, prompted us to find an optimized score from biomarkers available in the present cohort in an unsupervised manner. An ensemble biomarker discovery approach, which included several machine learning algorithms, identified age, γGT, HbA1c, M30, and adiponectin as ideal available parameters. The logistic regression model generated from these parameters has a strong correlation with NAS in the training and in the independent validation cohort. In addition, it demonstrated reasonable performance to discriminate between NAFL and NASH in both cohorts. Notably individual components of the new score are not limited to classic liver related markers. The selected markers reflect an overall metabolic health state:

- age as surrogate for the duration of metabolic derangements or obesity;
- γGT as classic liver injury marker;
- HbA1c as surrogate marker for impaired glucose metabolism;
- M30 as marker for epithelial / hepatocyte apoptosis;
- Adiponectin as marker for adipose tissue function.

We believe that this combination of factors from adipose tissue, glucose metabolism, and liver health to detect NASH represent processes influencing severity of NAFLD by altered hepatic metabolism in obesity. Of note, these markers have been selected by an unbiased biomarker discovery method and seem to reflect an organism wide metabolic situation, as shown in the cohort under weight loss therapy. One advantage of the new score is a continuous distribution of values. We currently refrain from giving a fixed cut off to exclude or definitely confirm NASH, as these may depend on the tested cohort. However, the score reflects a biological spectrum from low to high liver injury due to NAFLD.

In a cohort with treatment for obesity, the new score seems to allow assessment of the metabolic status of a patient. Higher scores demonstrate an impaired situation regarding metabolic syndrome and associated diseases, while negative values suggest a low risk to suffer from metabolic alterations. For an individual patient reduction of the score would demonstrate an improved situation and therapeutic benefit beyond mere weight loss. In parallel to weight loss and improvement of many health indicators, the new score dropped in 60% of the tested patients. Upon analysis of the remaining 40%, relatively low absolute weight loss, lower reduction of waist circumference, and only mildly impaired metabolic rate were associated with increased score values. This suggests that the new score is reflective of an overall metabolic health and could indicate objective efficiency of weight loss programs for metabolic health. This particular finding is of course limited by lack of liver biopsies as confirmation of the situation in the liver itself. Moreover, the baseline score differed significantly between the groups, suggesting that part of the study cohort did only have mild NAFLD related liver injury, which could not improve during the weight loss treatment. These and other possible confounders for monitoring with the new score need to be evaluated in appropriate study settings.

There are some limitations of the presented score. The training cohort for score generation was extremely obese, which might influence distribution of values for most of the included parameters. This includes a very low proportion of advanced fibrosis (2.4%), a common observation in extremely obese NAFLD patients [19,35]. Advanced fibrosis is the central determining factor for hepatic outcomes, including those in NAFLD, and for further clinical and therapeutic management [23,40,41]. Thus, non-invasive detection of advanced fibrosis would be more important from a mere hepatological point of view. As the new score was able to separate no or mild fibrosis from advanced fibrosis in this cohort, despite the low proportion of advanced fibrosis, it would be interesting to test the score in cohorts with higher prevalence. As limitation could also be seen that categorization of NAFL vs. NASH was performed by NAS [24], since this is still widely applied in clinical practice. Separation of NAFL vs. NASH by the SAF [11] would result in a slightly different score, nevertheless performance of the new score in the training cohort when SAF was applied was still reasonable. Another limitation is the population background that is mostly Caucasian/European. Data from other cohorts will be required to test whether the newly generated score can reach similar performance in other populations. To this end, this new score is freely available and open to use for testing in any cohort. Integration of new data into the score will hopefully refine it and further improve performance for specific cohorts and populations. One addition could be ultrasonographic or elastographic measurements, when available in a sufficiently large cohort. Finally, the use of adiponectin as parameter could be seen as another limitation, as it is not routinely measured, not even in settings of metabolic alterations, diabetes, or suspected NAFLD. It has been consistently shown that reduced adiponectin is closely associated to the status of the adipose tissue and the liver in obesity and metabolic syndrome [9,10,42–46]. Adiponectin also seems to indicate severity of glucose intolerance or insulin resistance and could possibly replace the BMI in screening approaches for diabetes [9,47,48]. In the present study, an unsupervised selection of parameters again resulted in adiponectin as one important factor indicating severity of liver injury in NAFLD. Thus, we deem it is about time to integrate adiponectin quantification into clinical routine for metabolic syndrome, insulin resistance, and associated diseases.

In summary, we present a new score for non-invasive assessment of the severity of NAFLD. Advantages of this score are a continuous distribution allowing disease assessment apart from a dichotomous classification as NAFL or NASH. Additional parameters, i.e., transient elastography or controlled attenuation parameter, could be added, given sufficiently large reference datasets. The score could possibly be used to monitor disease progression or resolution over time. We invite the scientific community and in particular all hepatologists examining patients suspected with NAFLD to test and apply the new score to assess patient health at http://CHek.heiderlab.de.

## Supporting information

S1 Fig Current non-invasive scores for assessment of NAFLD severity do not correlate with NAS. In a cohort of 164 obese individuals with NAFLD known non-invasive scores were tested for correlation with the NAS. The APRI (A) and Gholam’s score (B) achieved a reasonable Spearman r of 0.34 and 0.37, respectively. Though, neither the BARD score (C) nor the NAFLD fibrosis score (D), nor Palekars Score (E) correlated with the NAS in this cohort.
(JPG)

S2 Fig Current non-invasive scores cannot differentiate histological NASH from NAFL. In a cohort of 164 obese individuals with NAFLD AUCs were calculated to assess classification into NAFL or NASH by known non-invasive scores. The APRI score reached an AUC of 0.65 (A) and Gholam’s Score an AUC of 0.7 (B), both significantly better than random guessing. This was not the case for the BARD (C), the NAFLD fibrosis score (D), or Palekars Score (E).
(JPG)

S3 Fig Current non-invasive scores for assessment of NAFLD severity do not correlate with fibrosis stage. In a cohort of 164 obese individuals with NAFLD known non-invasive scores were tested for correlation with the fibrosis stage. None of the tested scores, APRI (A), Gholam’s Score (B), BARD (C), NAFLD fibrosis score (D), or Palekars score (E) were correlated to the histological fibrosis stage. ROC calculations did not show separation between no or mild fibrosis (grade 0–2) and advanced fibrosis (grades 3–4), that would have been better than random guessing (not shown). This lack of performance might be due to the very low number of individuals with advanced fibrosis (Grade 3: n = 3; grade 4: n = 1).
(JPG)

S4 Fig The new score significantly correlates with SAF score. Using NAS-based classification of NAFLD to generate the new score might be seen as limitation. Thus the score was correlated to the SAF in the training (A) and validation cohort (C), resulting in significant robust correlations. In addition the score was tested to separate NAFL from NASH according to the Bedossa algorithm (at least 1 point in steatosis, ballooning and lobular inflammation each to diagnose NASH). The new score achieved reasonable performance in the training cohort (B) but insufficient performance in the validation cohort (D), though still significant versus random guessing.
(JPG)

S1 Table Significant correlations of the new score with available parameters in a subgroup of the lifestyle intervention cohort at T0. (PDF)

S2 Table Significant correlations of the new score with available parameters in a subgroup of the lifestyle intervention cohort at T52. (PDF)

S3 Table Raw data tables for all datasets. (XLSX)
