# Supplementary figures and images for: Non-invasive assessment of NAFLD as systemic disease—A machine learning perspective
Source: PLoS One. 2019 Mar 26;14(3):e0214436. doi: 10.1371/journal.pone.0214436 (PMC6435145; doi:10.1371/journal.pone.0214436)

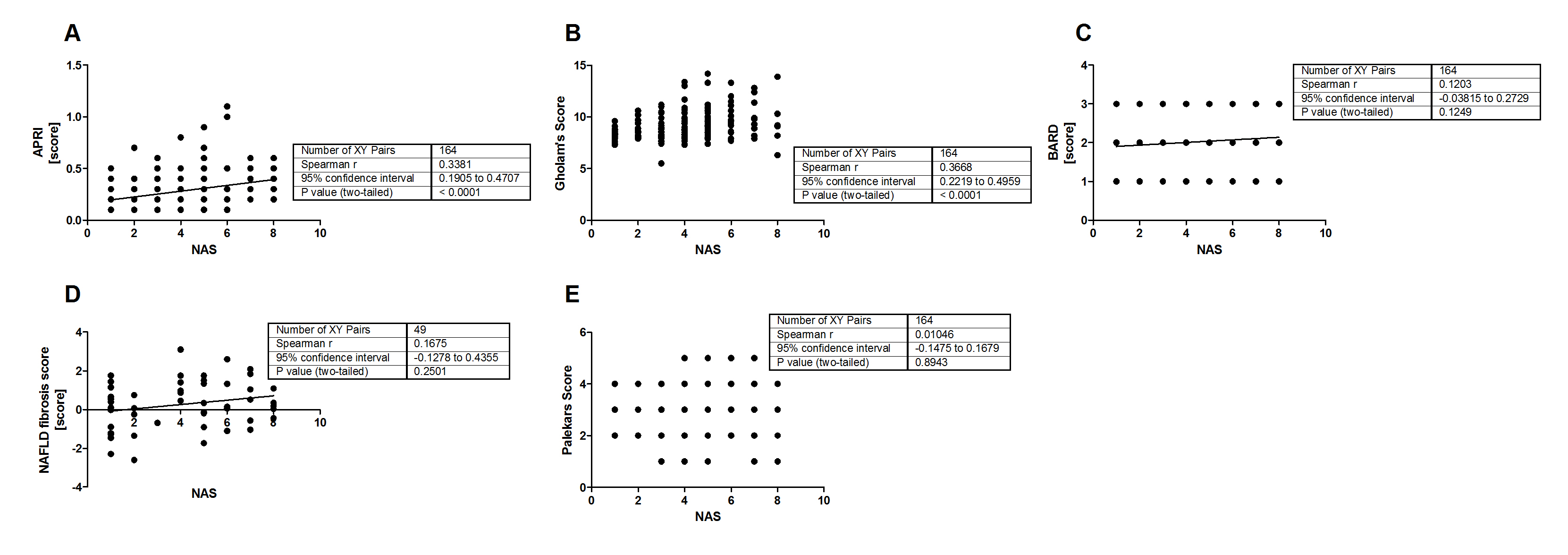

Supplement: S1 Fig — In a cohort of 164 obese individuals with NAFLD known non-invasive scores were tested for correlation with the NAS. The APRI (A) and Gholam’s score (B) achieved a reasonable Spearman r of 0.34 and 0.37, respectively. Though, neither the BARD score (C) nor the NAFLD fibrosis score (D), nor Palekars Score (E) correlated with the NAS in this cohort. (JPG) [file pone.0214436.s001.jpg]

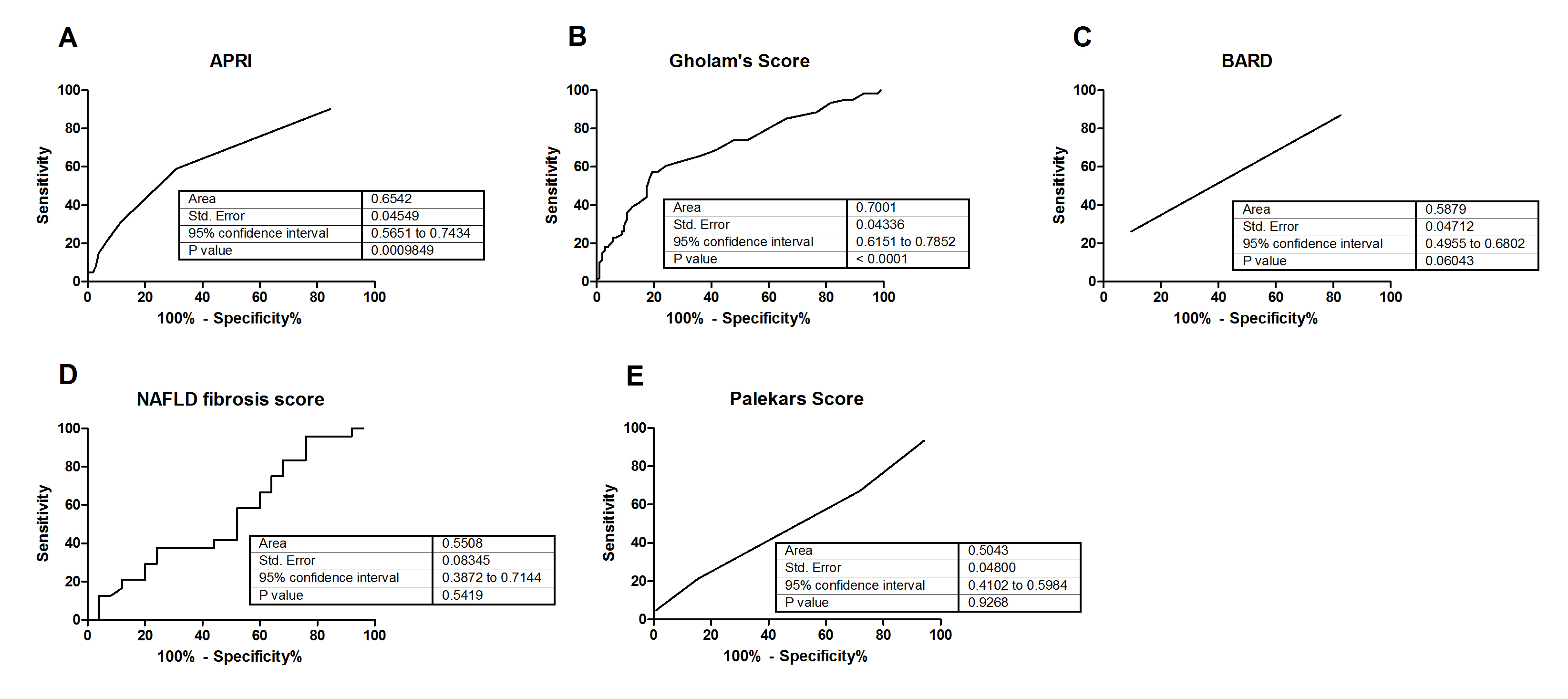

Supplement: S2 Fig — In a cohort of 164 obese individuals with NAFLD AUCs were calculated to assess classification into NAFL or NASH by known non-invasive scores. The APRI score reached an AUC of 0.65 (A) and Gholam’s Score an AUC of 0.7 (B), both significantly better than random guessing. This was not the case for the BARD (C), the NAFLD fibrosis score (D), or Palekars Score (E). (JPG) [file pone.0214436.s002.jpg]

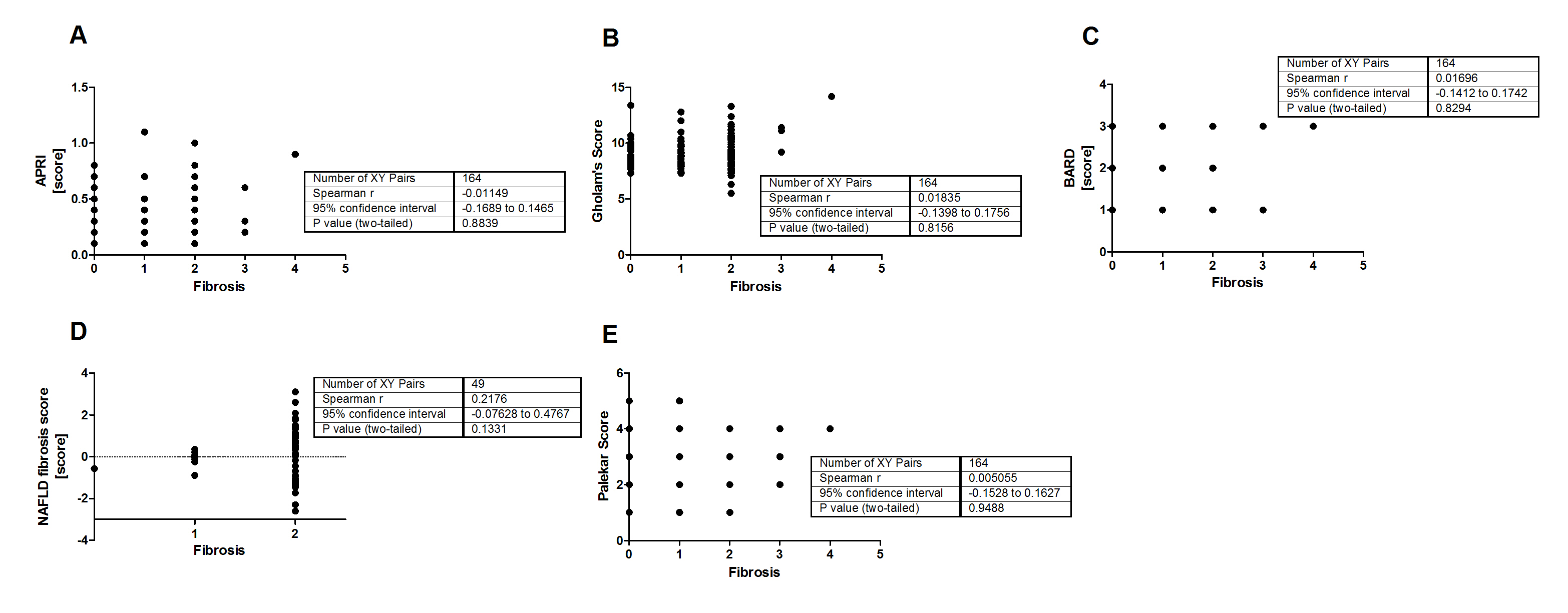

Supplement: S3 Fig — In a cohort of 164 obese individuals with NAFLD known non-invasive scores were tested for correlation with the fibrosis stage. None of the tested scores, APRI (A), Gholam’s Score (B), BARD (C), NAFLD fibrosis score (D), or Palekars score (E) were correlated to the histological fibrosis stage. ROC calculations did not show separation between no or mild fibrosis (grade 0–2) and advanced fibrosis (grades 3–4), that would have been better than random guessing (not shown). This lack of performance might be due to the very low number of individuals with advanced fibrosis (Grade 3: n = 3; grade 4: n = 1). (JPG) [file pone.0214436.s003.jpg]

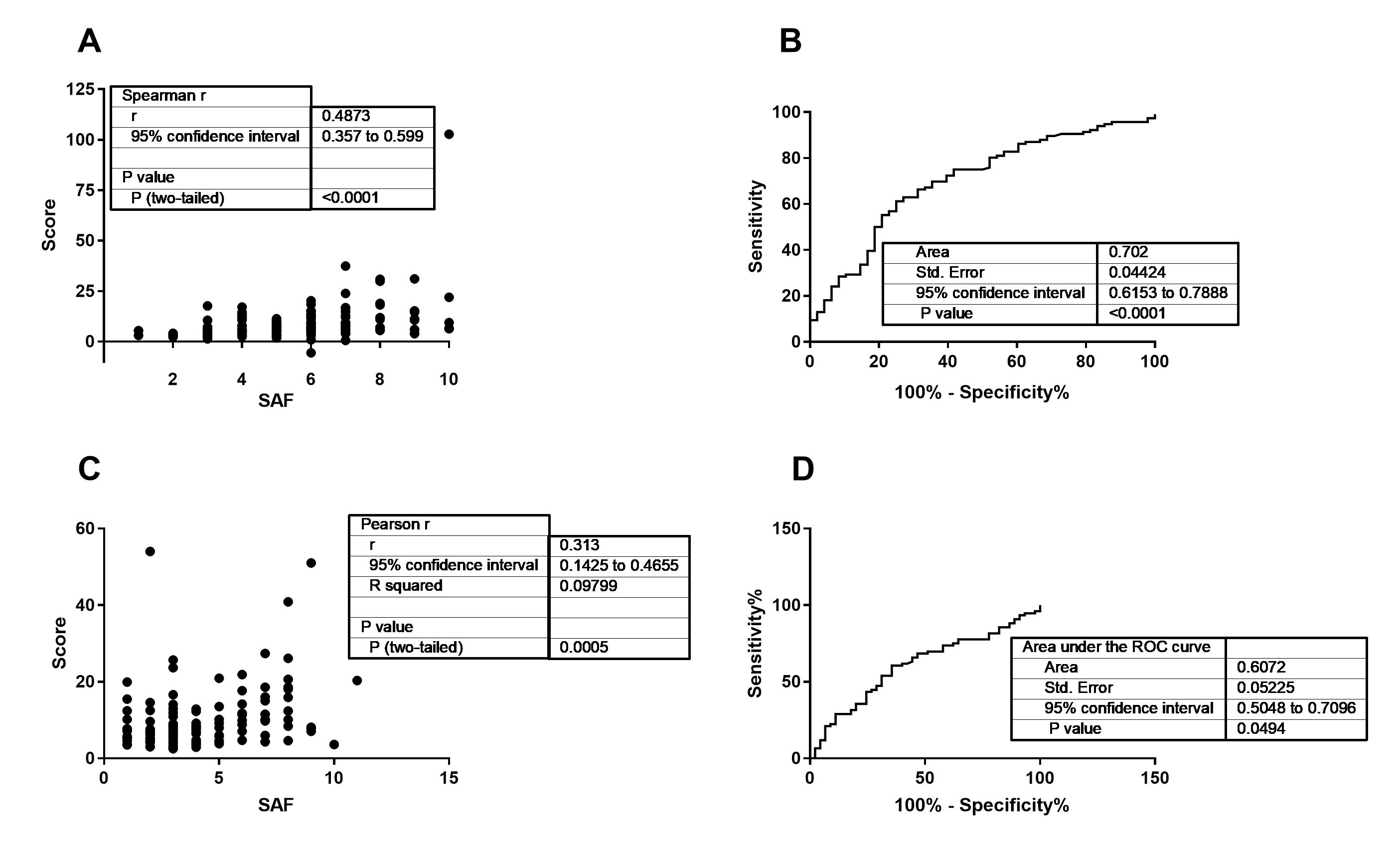

Supplement: S4 Fig — Using NAS-based classification of NAFLD to generate the new score might be seen as limitation. Thus the score was correlated to the SAF in the training (A) and validation cohort (C), resulting in significant robust correlations. In addition the score was tested to separate NAFL from NASH according to the Bedossa algorithm (at least 1 point in steatosis, ballooning and lobular inflammation each to diagnose NASH). The new score achieved reasonable performance in the training cohort (B) but insufficient performance in the validation cohort (D), though still significant versus random guessing. (JPG) [file pone.0214436.s004.jpg]
