# Supplementary material for: Non-invasive assessment of NAFLD as systemic disease—A machine learning perspective
Source: PLoS One. 2019 Mar 26;14(3):e0214436. doi: 10.1371/journal.pone.0214436 (PMC6435145; doi:10.1371/journal.pone.0214436)
Supplement: S1 Table — (PDF) [file pone.0214436.s005.pdf]

Supplementary Table 1: Significant correlations of the new score with available parameters in a subgroup of the lifestyle intervention cohort at T0.

Parameter	Number of Pairs	Spearman r	95% confidence interval	P value
Sex	38	0.4207	0.1069 to 0.6582	0.0085
Phase angle	38	0.3645	0.04086 to 0.6189	0.0245
Basal metabolic rate	38	0.4503	0.1429 to 0.6785	0.0046
ECM/BCM-Index	38	-0.3657	-0.6198 to -0.04226	0.024
Body fat %	38	-0.3899	-0.6369 to -0.07040	0.0155
R (bioelectrical impedance analysis)	38	-0.4175	-0.6560 to -0.1031	0.0091
AST	36	0.372	0.03931 to 0.6304	0.0255
HDL	38	-0.4433	-0.6737 to -0.1343	0.0053
Hb	38	0.3849	0.06458 to 0.6334	0.017
Ht %	38	0.3855	0.06527 to 0.6338	0.0168
Waist circumference	38	0.4037	0.08665 to 0.6465	0.012
Waist/Hip ratio	38	0.4607	0.1558 to 0.6855	0.0036
Fatty liver index	38	0.473	0.1711 to 0.6937	0.0027
