# Supplementary material for: Non-invasive assessment of NAFLD as systemic disease—A machine learning perspective
Source: PLoS One. 2019 Mar 26;14(3):e0214436. doi: 10.1371/journal.pone.0214436 (PMC6435145; doi:10.1371/journal.pone.0214436)
Supplement: S2 Table — (PDF) [file pone.0214436.s006.pdf]

Supplementary Table 2: Significant correlations of the new score with available parameters in a subgroup of the lifestyle intervention cohort at T52.

Parameter	Number of Pairs	Spearman r	95% confidence interval	P value
Steatosis in ultrasound	28	0.4388	0.06700 to 0.7036	0.0195
Phase angle	38	0.325	-0.003902 to 0.5905	0.0465
Basal metabolic rate	38	0.4012	0.08374 to 0.6448	0.0125
R (bioelectrical impedance analysis)	38	-0.4035	-0.6463 to -0.08638	0.012
AST	38	0.3688	0.04582 to 0.6220	0.0227
ALT	38	0.4913	0.1941 to 0.7059	0.0017
Uric acid	38	0.4299	0.1181 to 0.6646	0.0071
Serum triglycerides	38	0.3803	0.05917 to 0.6301	0.0185
HDL	38	-0.4729	-0.6936 to -0.1709	0.0027
Waist circumference	37	0.4377	0.1226 to 0.6726	0.0067
Waist/Hip ratio	37	0.4197	0.1009 to 0.6604	0.0097
Ghrelin	38	-0.4409	-0.6720 to -0.1314	0.0056
Fatty liver index	38	0.438	0.1279 to 0.6701	0.006
